# The Interplay between Maternal and Post-Weaning High-Fat Diet and Gut Microbiota in the Developmental Programming of Hypertension

**DOI:** 10.3390/nu11091982

**Published:** 2019-08-22

**Authors:** Chien-Ning Hsu, Chih-Yao Hou, Chien-Te Lee, Julie Y.H. Chan, You-Lin Tain

**Affiliations:** 1 Department of Pharmacy, Kaohsiung Chang Gung Memorial Hospital, Kaohsiung 833, Taiwan; 2 School of Pharmacy, Kaohsiung Medical University, Kaohsiung 807, Taiwan; 3 Department of Seafood Science, National Kaohsiung University of Science and Technology, Kaohsiung 811, Taiwan; 4 Division of Nephrology, Kaohsiung Chang Gung Memorial Hospital, Kaohsiung 833, Taiwan; 5 Institute for Translational Research in Biomedicine, Kaohsiung Chang Gung Memorial Hospital and Chang Gung University College of Medicine, Kaohsiung 833, Taiwan; 6 Department of Pediatrics, Kaohsiung Chang Gung Memorial Hospital and Chang Gung University College of Medicine, Kaohsiung 833, Taiwan

**Keywords:** AMP-activated protein kinase, butyrate, developmental origins of health and disease (DOHaD), gut microbiota, high fat diet, hypertension, nutrient-sensing signals, propionate, short chain fatty acids

## Abstract

Excessive intake of saturated fat has been linked to hypertension. Gut microbiota and their metabolites, short-chain fatty acids (SCFAs), are known to be involved in the development of hypertension. We examined whether maternal and post-weaning high-fat (HF) diet-induced hypertension in adult male offspring is related to alterations of gut microbiota, mediation of SCFAs and their receptors, and downregulation of nutrient-sensing signals. Female Sprague–Dawley rats received either a normal diet (ND) or HF diet (D12331, Research Diets) during pregnancy and lactation. Male offspring were put on either the ND or HF diet from weaning to 16 weeks of age, and designated to four groups (maternal diet/post-weaning diet; *n* = 8/group): ND/ND, HF/ND, ND/HF, and HF/HF. Rats were sacrificed at 16 weeks of age. Combined HF/HF diets induced elevated blood pressure (BP) and increased body weight and kidney damage in male adult offspring. The rise in BP is related to a downregulated AMP-activated protein kinase (AMPK)–peroxisome proliferator-activated receptor co-activator 1α (PGC-1α) pathway. Additionally, HF/HF diets decreased fecal concentrations of propionate and butyrate and decreased G protein-coupled receptor 41 (GPR41), but increased olfactory receptor 78 (Oflr78) expression. Maternal HF diet has differential programming effects on the offspring’s microbiota at 3 and 16 weeks of age. Combined HF/HF diet induced BP elevation was associated with an increased *Firmicutes* to *Bacteroidetes* ratio, increased abundance of genus *Akkermansia* and phylum *Verrucomicrobia*, and reduced abundance in genus *Lactobacillus*. Maternal gut microbiota-targeted dietary interventions might be reprogramming strategies to protect against programmed hypertension in children and their mothers on consumption of a fat-rich diet.

## 1. Introduction

Non-communicable diseases (NCDs) are increasingly becoming the leading causes of global morbidity and mortality [1]. Among NCDs, hypertension-related diseases are the most common causes of deaths. Despite substantial advances in therapy, the global epidemic rise of NCDs remains a significant challenge. Early-life exposure can program the onset of chronic NCDs [2], now framed as the “developmental origins of health and disease” (DOHaD) [3]. 

Perinatal nutrition affects fetal development and long-term health of the offspring. Imbalanced maternal diet may induce fetal programming that permanently alters the morphology and function of fetal organs and systems, leading to various NCDs, including hypertension [4]. The high-fat (HF) diet model has been used to study obesity-related disorders like hypertension [5,6]. Along these lines, using a rat model of maternal plus post-weaning HF diets, we have demonstrated that adult male offspring exposed to HF intake develop hypertension [7]. 

Among the proposed mechanisms linking maternal nutritional insults to offspring adverse outcomes, changes of gut microbiota and their metabolites have recently received more attention [4,8]. Diet is an instrumental factor in shaping the gut microbiota. Increasing evidence links gut microbiota dysbiosis to the development of a variety of diseases [9]. During pregnancy, the diet–gut microbiota interactions can mediate epigenetic regulation of gene expression not only in mother but also in the fetus via the contact with their metabolites [10]. The offspring gut microbiota is highly sensitive to the early-life environmental stimuli. Accordingly, maternal diet can influence the gut microbiota of mothers and their offspring, consequently driving developmental programming of chronic diseases in adult offspring [11,12]. Although several microbial markers have been reported related to HF consumption, like increased abundance of phylum *Firmicutes* and decreased *Bacteroidetes* [9], whether a similar pattern of results can be obtained from offspring born to mothers fed with HF diet is largely unknown.

The gut microbiota produces a variety of metabolites like short-chain fatty acids detectable in host circulation [13]. Short-chain fatty acids (SCFAs, e.g., acetate, butyrate, and propionate) and their receptors are reported to be involved in the regulation of blood pressure (BP) [14]. In line with this, a recent study from our laboratory reported that prebiotic or probiotic therapy can alter gut microbiota, regulate SCFAs and their receptors, and mediate nutrient-sensing signals to protect adult male offspring against hypertension programmed by high-fructose diet [15]. 

Nutrient-sensing signals are regarded as key players in the developmental programming of hypertension, such as 5’-adenosine monophosphate-activated protein kinase (AMPK), peroxisome proliferator-activated receptor (PPAR), and PPARγ co-activator 1α (PGC-1α) [4,16]. Activation of AMPK by resveratrol can affect PGC-1α activity to regulate the downstream expression of PPAR target genes [17]. Our recently published study demonstrated that HF diet-induced hypertension is correlated to inhibitory AMPK/PGC-1α pathway and altered gut microbiota [18].

Our objective in this study was to examine whether maternal and post-weaning HF diet cause differential effects on BP, gut microbiota, SCFAs and their receptors, and nutrient-sensing signals in adult offspring. 

## 2. Materials and Methods 

### 2.1. Animal Model 

This study was followed the Guide for the Care and Use of Laboratory Animals of the National Institutes of Health. The protocol was approved by the Institutional Animal Care and Use Committee of the Kaohsiung Chang Gung Memorial Hospital (IACUC permit number: 201721408). Virgin female Sprague–Dawley (SD) rats (*n* = 12) were obtained from BioLASCO Taiwan Co., Ltd. (Taipei, Taiwan) and maintained in an Association for Assessment and Accreditation of Laboratory Animal Care International (AAALAC)-approved animal facility in our hospital. The rats were housed in a in a controlled environment with 12:12 light-dark cycle and humidity of 55%, throughout the study. Male SD rats were caged with female rats until mating. The presence of the plug confirmed mating. Female rats were weight-matched and assigned to receive either a normal diet with regular rat chow (ND; Fwusow Taiwan Co., Ltd., Taichung, Taiwan; 52% carbohydrates, 23.5% protein, 4.5% fat, 10% ash, and 8% fiber) or a 58% high-fat diet (D12331, Research Diets, Inc., New Brunswick, NJ, USA; 58% fat (hydrogenated coconut oil), 25.5% carbohydrate, 16.4% protein, and 0% fiber) during pregnancy and lactation. After birth, litters were culled to eight from each mother to standardize the received quantity of milk and maternal pup care. Since men are much more likely to be hypertensive than women at a younger age [19], only male offspring were used. Male offspring were weaned at 3 weeks of age, and onto either the normal diet (ND) or HF diet ad libitum from weaning to 16 weeks of age. Rats were assigned to four experimental groups (maternal diet/postweaning diet; *n* = 8/group): ND/ND, HF/ND, ND/HF, and HF/HF. 

We used BP-2000 tail-cuff system (BP-2000, Visitech Systems, Inc., Apex, NC, USA) to measure BP in conscious rats at 3, 4, 8, 12, and 16 weeks of age [7]. Rats were allowed to adapt to restraint and tail-cuff inflation for 1 week prior to the experiment. Rats were placed on the specimen platform. Their tails were passed through a cuff and immobilized by adhesive tape. Following a 10-min warm-up period, 10 preliminary cycles were performed to allow the rats to adjust to the inflating cuff. For each rat, three stable measures were taken and averaged. Fresh feces samples were collected at 3 and 16 weeks of age, frozen, and stored at −80 °C until use. At 16 weeks of age, rats were anesthetized by intraperitoneally injecting ketamine (50 mg/kg body weight) and xylazine (10 mg/kg body weight) and were euthanized by intraperitoneally injecting an overdose of pentobarbital for sacrifice. Blood samples were collected. Kidneys were harvested and stored at −80 °C in a freezer for further analysis. 

### 2.2. Gas Chromatography-Flame Ionization Detector (GC-FID) 

We used gas chromatography-mass spectrometry (GCMS-QP2010; Shimadzu, Kyoto, Japan) with a flame ionization detector (FID) to measure levels of acetate, butyrate, and propionate in the plasma and feces [15]. We used internal standards in analytical standard grades for acetate and propionate (from Sigma-Aldrich, St. Louis, MO, USA), and for butyrate (from Chem Service, West Chester, PA, USA). The working solutions of used as internal and external standards were prepared at the concentration of 10 mM. These solutions were kept at −20 °C in a freezer. Dry air, nitrogen, and hydrogen were supplied to the FID at 300, 20 and 30 mL/min, respectively. A 2-µL aliquot of sample was injected into the column. The inlet and FID temperature were set at 200 and 240 °C, respectively. The total running time was 17.5 min.

### 2.3. Analysis of Gut-Microbiota Composition

Metagenomic DNA was extracted from frozen fecal samples after centrifugation. All polymerase chain-reaction amplicons were mixed together and sent to the Genomic and Proteomic Core Laboratory, Kaohsiung Chang Gung Memorial Hospital (Kaohsiung, Taiwan) for sequencing using an Illumina Miseq platform (Illumina, San Diego, CA, USA) [15]. Amplicons were prepared according to the 16S Metagenomics Sequencing Library Preparation protocol (Illumina, San Diego, CA, USA), and sequenced with the Illumina MiSeq platform (Illumina, San Diego, CA, USA). Sequences (Illumina, San Diego, CA, USA) with a distance-based similarity of 97% or greater were grouped into operational taxonomic units (OTUs) using the USEARCH algorithm. To determine the significantly differential taxa, we applied linear discriminant analysis effect size (LEfSe) to compare samples between groups. The LEfSe uses linear discriminant analysis (LDA) to estimate the effect size of each differentially abundant feature. The threshold of the linear discriminant was set to two.

### 2.4. Western Blot

Western blot analysis was performed using the methods published previously [7]. Protein samples (200-μg kidney cortex) were boiled with gel-loading buffer for 5 min, subjected to 10–15% SDS-PAGE, and then transferred to a nitrocellulose membrane (GE Healthcare Bio-Sciences Corp., Piscataway, NJ, USA). To verify equal loading, the membranes were incubated with Ponceau S red (PonS) stain solution (Sigma-Aldrich, St. Louis, MO, USA) for 10 min on the rocker. Two nutrient-sensing signals, AMPKα2 and PGC-1α, were analyzed. Additionally, we determined the protein abundance of three SCFA receptors, including G protein-coupled receptor 41 (GPR41), GPR43, and olfactory receptor 78 (Olfr78). We used the following primary antibodies: a rabbit polyclonal anti-rat phosphorylated AMPKα1/2 antibody (1:1000, overnight incubation; Santa Cruz Biotechnology), a rabbit polyclonal anti-PGC-1α antibody (1:1000, overnight incubation; Abcam, Cambridge, MA, USA), a rabbit polyclonal anti-GPR41 antibody (1:500, overnight incubation; USBiological, Salem, MA, USA), a rabbit polyclonal anti-GPR43 antibody (1:500, overnight incubation; Millipore, Burlington, MA, USA), and a rabbit polyclonal anti-Olfr78 antibody (1:500, overnight incubation; Assay Biotech, Fremont, CA, USA). Next, the membrane was washed five times with 0.1% T-TBS, incubated for 1h with a peroxidase-labeled secondary antibody diluted 1:1000 in T-TBS, and then developed using Chemi Doc (Bio-rad Image Lab 5.0). Bands were quantified by densitometry as integrated optical density (IOD). IOD was then normalized to total protein PonS staining. The protein abundance was represented as IOD/PonS.

### 2.5. Immunohistochemistry Staining

Paraffin-embedded tissues sectioned at 3-μm thickness were deparaffinized in xylene and rehydrated in a graded ethanol series to phosphate-buffered saline. Following blocking with immunoblock (BIOTnA Biotech., Kaohsiung, Taiwan), the sections were incubated for 2 h at room temperature with an anti-phosphorylated AMPKα2 antibody (1:400, Cell Signaling, Danvers, MA, USA) or an anti-PGC-1α antibody (1:200, Abcam, Cambridge, MA, USA). Immunoreactivity was revealed using the polymer-horseradish peroxidase (HRP) labeling kit (BIOTnA Biotech). For the substrate–chromogen reaction, 3,30-diaminobenzidine (DAB) was used. An identical staining protocol omitting incubation with primary antibody was employed to prepare samples that were used as negative controls. Renal cells positive for immunostaining were examined in 10 randomly selected ×400 microscopic fields per section. The number of immunostained cells was expressed as we described previously [18].

### 2.6. Statistical Analysis 

Data are reported as the mean ± standard error of mean (SEM). A value of *p* < 0.05 was considered statistically significant. Statistical analysis was conducted with one-way analysis of variance (ANOVA) with a Tukey post hoc test for multiple comparisons. Analyses were performed using the SPSS software 14.0 (SPSS Inc., Chicago, IL, USA).

## 3. Results

### 3.1. The Effects of Maternal and Post-Weaning HF Diet on Morphological Values and BPs

**Figure 1:**
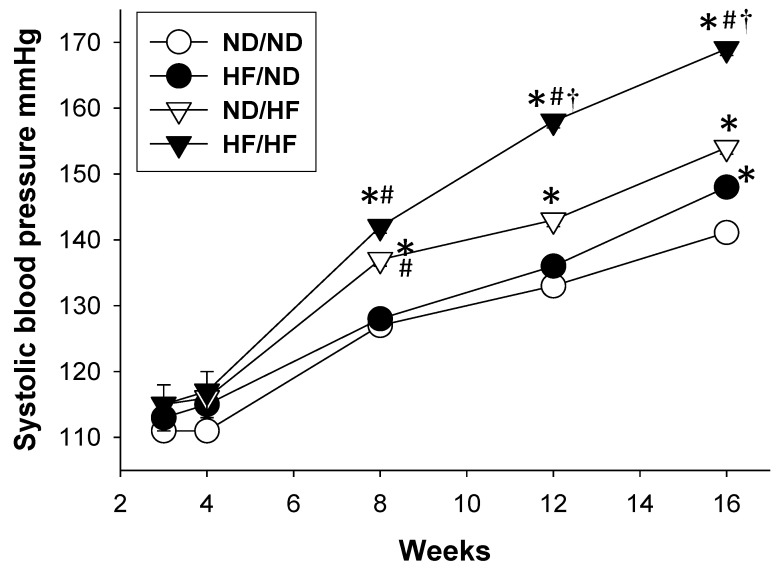
Effects of maternal and postnatal high-fat (HF) diet on systolic blood pressure in male offspring from 3 to 16 weeks. ND/ND, maternal plus post-weaning normal diet; HF/ND, maternal high-fat diet plus post-weaning normal diet; ND/HF, maternal normal diet plus post-weaning high-fat diet; HF/HF, maternal plus post-weaning high-fat diet. * *p* < 0.05 vs. ND/ND; # *p* < 0.05 vs. HF/ND; † *p* < 0.05 vs. ND/HF.

Post-weaning consumption of HF diet caused a greater body weight (BW) compared with controls and the HF/ND group, with the greatest BW in the HF/HF group (Table 1). The kidney weights and the ratios of kidney weight-to-body weight were lower in the ND/HF and HF/HF groups compared to controls and the HF/ND groups. At 16 weeks of age, maternal and post-weaning HF diet increased systolic BP by 5 and 11 mmHg compared to controls, respectively. There is a synergistic effect of maternal and post-weaning HF diet on systolic BP, resulting in an increase of ~26 mmHg in the HF/HF group versus control. Similarly, diastolic BP and mean arterial pressure were higher in the HF/ND and ND/HF group compared with those in the control group, with the highest in the HF/HF group. Figure 1 shows the systolic BPs of ND/HF and HF/HF group were significantly higher than those in the control group from 8 to 16 weeks. By 12 weeks of age, the systolic BP had significantly increased in the HF/HF group relative to the other three groups. The plasma creatinine level was higher in HF/HF group compared to the controls. These findings indicate that maternal or post-weaning HF diet more or less caused a rise in BW and BPs, which was enhanced to a greater extent in the combined HF/HF diets. However, only combined HF/HF diet resulted in kidney damage, represented by elevated creatinine levels.

### 3.2. The Effects of Maternal and Post-Weaning HF Diet on Nutrient-Sensing Signals

**Figure 2:**
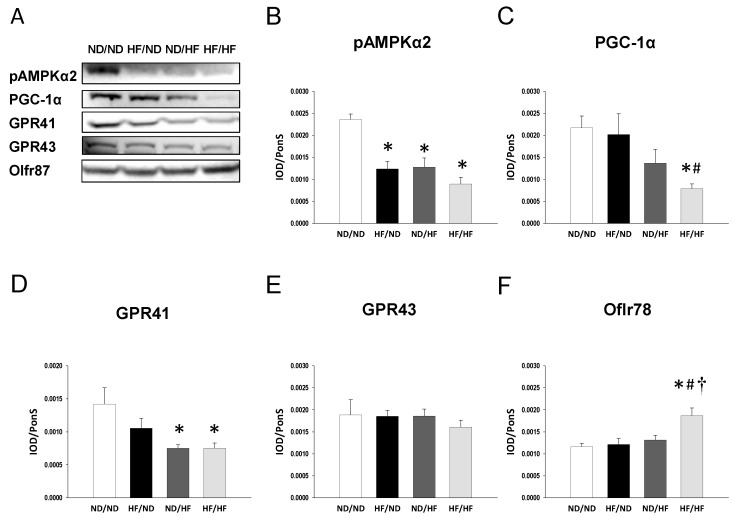
(**A**) Representative western blots showing phosphorylated AMP-activated protein kinase (AMPKα2, ~63kDa), peroxisome proliferator-activated receptor co-activator 1α (PGC-1α, ~90kDa), G protein-coupled receptor 41 (GPR41, ~38kDa), GPR43 (~47kDa), and olfactory receptor 78 (Oflr78) (~35kDa) bands in offspring kidneys at 16 weeks of age. Relative abundance of renal cortical (**B**) phosphorylated AMPKα2, (**C**) PGC-1α, (**D**) GPR41, (**E**) GPR43, and (**F**) Oflr78 were quantified. ND/ND, maternal plus post-weaning normal diet; HF/ND, maternal high-fat diet plus post-weaning normal diet; ND/HF, maternal normal diet plus post-weaning high-fat diet; HF/HF, maternal plus post-weaning high-fat diet. *n* = 8/group. * *p* < 0.05 vs. ND/ND; # *p* < 0.05 vs. HF/ND; † *p* < 0.05 vs. ND/HF.

**Figure 3:**
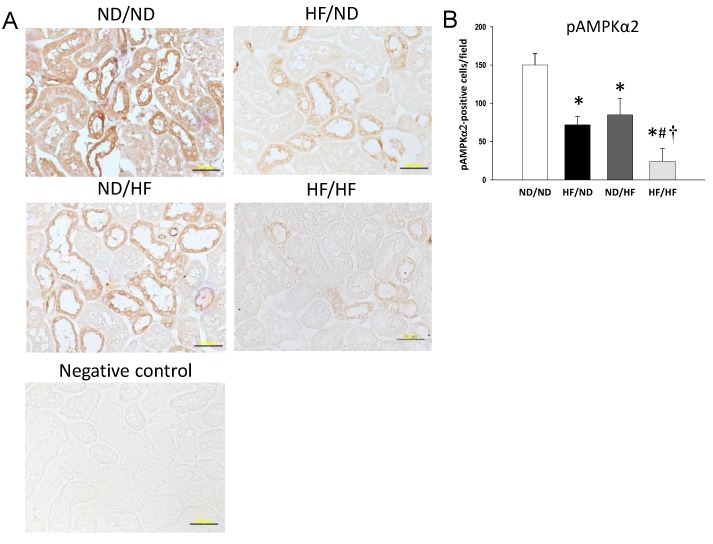
(**A**) Light microscopic findings of phosphorylated AMPKα2 immunostaining in the kidney cortex in 16-week-old male offspring. Bar = 50 μm; (**B**) Quantitative analysis of phosphorylated AMPKα2-positive cells per microscopic field (400×); * *p* < 0.05 vs. ND/ND; # *p* < 0.05 vs. HF/ND; † *p* < 0.05 vs. ND/HF.

**Figure 4:**
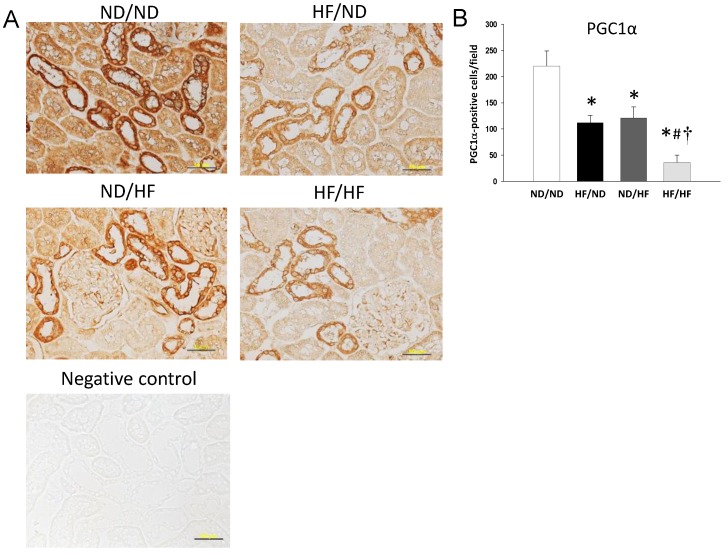
(**A**) Light microscopic findings of PGC-1α immunostaining in the kidney cortex in 16-week-old male offspring. Bar = 50 μm; (**B**) Quantitative analysis of PGC-1α-positive cells per microscopic field (400×); * *p* < 0.05 vs. ND/ND; # *p* < 0.05 vs. HF/ND; † *p* < 0.05 vs. ND/HF.

We evaluated key elements in the nutrient-sensing pathway, including phosphorylated AMPKα2 and PGC-1α. As shown in Figure 2, the renal protein level of phosphorylated AMPKα2 (Figure 2B) was lower in the HF/ND, ND/HF, and HF/HF group compared with that in the ND/ND group. Additionally, the HF/HF diet caused a significant reduction of PGC-1α versus the controls in offspring kidneys (Figure 2C). We next evaluated phosphorylated AMPKα2 (Figure 3) and PGC-1α (Figure 4) in the offspring kidneys by immunohistochemistry. 

Immunostaining of phosphorylated AMPKα2 in the glomeruli and renal tubules indicated intense staining in the ND/ND group (150 ± 15 positive cells), an intermediate level of staining in the HF/ND group (72 ± 11 positive cells) and ND/HF group (85 ± 21 positive cells), and little staining in the HF/HF group (24 ± 17 positive cells) (Figure 3B). Similar to phosphorylated AMPKα2, maternal or post-weaning HF diet significantly decreased PGC-1α expression in the HF/ND group (112 ± 14 positive cells) and the ND/HF group (121 ± 21 positive cells) vs. the ND/ND group (220 ± 29 positive cells) (Figure 4A). Combined maternal and post-weaning HF diets caused the reduction of PGC-1α expression to a greater extent (36 ± 14 positive cells) (Figure 4B). Taken together, these findings indicated that HF/HF diet synergistically downregulated AMPK–PGC-1α pathway.

### 3.3. The Effects of Maternal and Post-Weaning HF Diet on SCFAs and Their Receptors

It was reported previously that SCFAs are involved in the development of hypertension [14]. We investigated whether HF diet causes a rise in BP is related to alterations of SCFAs production and the expression of SCFA receptors. Our results demonstrated that post-weaning HF diet decreased fecal concentrations of acetate compared to the ND/ND and HF/ND group (Table 2). Fecal propionate and butyrate levels were lower in the ND/HF group than those in the ND/ND and HF/ND group. Similarly, combined maternal and post-weaning HF reduced fecal concentrations of propionate and butyrate compared to controls. We next evaluated the protein levels of SCFA receptors. Renal GPR41 expression was lower in the ND/HF and HF/HF group compared to that in the ND/ND group (Figure 2D). GPR43 protein level in offspring kidney was not different among the four groups (Figure 2E). However, combined HF/HF diets resulted in a significant increase of renal Olfr78 expression compared to the other three groups (Figure 2F). 

### 3.4. The Effects of Maternal and Post-Weaning HF Diet on Gut Microbiota

**Figure 5:**
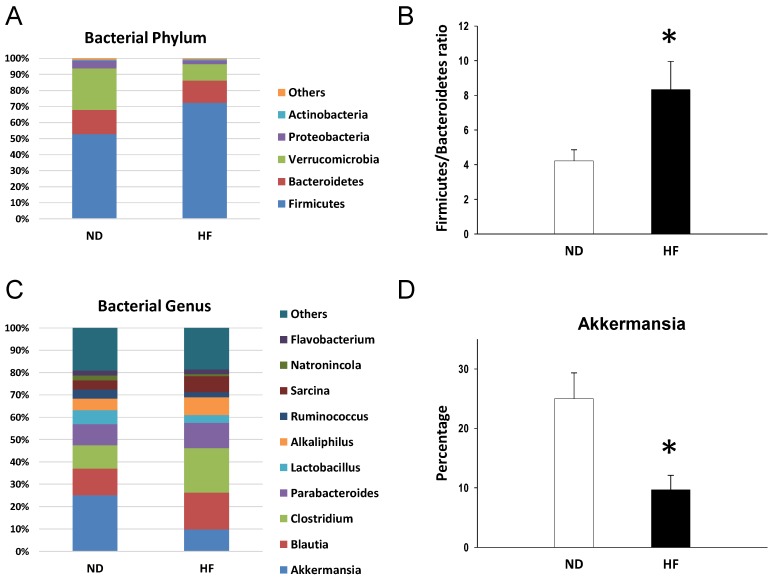
Effect of maternal high-fructose (HF) diet on offspring gut microbiota at 3 weeks of age. (**A**) Relative abundances of the top five phyla. (**B**) The *Firmicutes* to *Bacteroidetes* ratio. (**C**) Relative abundances of the top 10 genera. (**D**) Relative abundances of the genus *Akkermansia*. *n* = 16/group. * *p* < 0.05 vs. ND.

**Figure 6:**
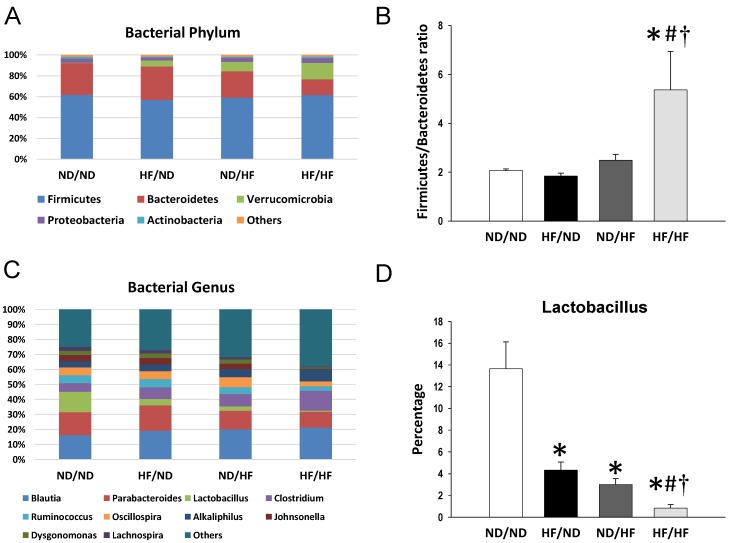
Effect of maternal and post-weaning high-fructose (HF) diet on offspring gut microbiota at 16 weeks of age. (**A**) Relative abundances of the top five phyla. (**B**) The *Firmicutes* to *Bacteroidetes* ratio. (**C**) Relative abundances of the top 10 genera. (**D**) Relative abundances of the genus *Lactobaccilus*. *n* = 8/group. * *p* < 0.05 vs. ND/ND; # *p* < 0.05 vs. HF/ND; † *p* < 0.05 vs. ND/HF.

We further analyzed bacterial populations in the gut at the phylum and genus levels at 3 weeks (Figure 5) and 16 weeks of age (Figure 6). At 3 weeks, the age of weaning, the main phyla in the offspring born of dams fed with regular chow (ND) or HF diet were *Firmicutes*, *Bacteroidetes*, *Verrucomicrobia*, *Proteobacteria*, and *Actinobacteria*. Maternal HF intake caused a remarkable increase in the phylum *Firmicutes* (72.3 ± 4% vs. 52.9 ± 3.7%; *p* = 0.002), but a decrease in the *Verrucomicrobia* (10.3 ± 2.6% vs. 25.9 ± 4.5%; *p* = 0.007) and *Proteobacteria* (2.4 ± 0.2% vs. 4.8 ± 0.4%; *p* < 0.001) (Figure 5A). The *Firmicutes* to *Bacteroidetes* ratio has been considered a signature for hypertension [20,21]. In the current study, the *Firmicutes* to *Bacteroidetes* ratio was higher in the HF group (8.3 ± 1.6) compared to that in the control group (4.2 ± 0.6, *p* = 0.03) (Figure 5B). Additionally, the main bacterial genera were *Akkermansia*, *Blautia*, *Clostridium*, *Parabacteroides*, *Lactobacillus*, *Alkaliphilus*, *Ruminococcus*, *Sarcina*, *Natronincola*, and *Flavobacterium* (Figure 5C). Among them, maternal HF diet decreased abundance of genus *Akkermansia* (9.7 ± 2.4% vs. 25 ± 4.3%; *p* = 0.006) (Figure 5D). Conversely, abundance of genus *Clostridium* was induced in the HF group (19.9 ± 2.6%) compared with that in control (10.4 ± 1.2%; *p* = 0.03). 

As shown in Figure 6A, the main phyla in the offspring gut microbiota at 16 weeks were identical to those at 3 weeks of age. Combined HF/HF diet significantly reduced the abundance of the phylum *Bacteroidetes* (15.1 ± 1.7% vs. 30.2 ± 0.7%; *p* < 0.001), while augmenting the abundance of the *Verrucomicrobia* (15.6 ± 2.2% vs. 0.5 ± 0.2%; *p* < 0.001). Additionally, the *Firmicutes* to *Bacteroidetes* ratio was the highest in the HF/HF group compared to that in the other three groups (All *p* < 0.05) (Figure 6B). 

Maternal HF intake decreased the abundance of genera *Lactobacillus* (HF/ND vs. ND/ND = 4.3 ± 0.8% vs. 13.7 ± 2.5%, *p* = 0.005) and *Turicibacter* (HF/ND vs. ND/ND = 0.9 ± 0.3% vs. 2.1 ± 0.4%, *p* = 0.035). The post-weaning HF diet caused an increase of genus *Akkermansia* (ND/HF vs. ND/ND = 9.4 ± 3.8% vs. 0.4 ± 0.2%, *p* = 0.002), and decreased the abundance of genera *Lactobacillus* (3 ± 0.5%, *p* = 0.002) and *Turicibacter* (0.6 ± 0.1%, *p* = 0.01). Combined HF/HF diet caused increases of several bacterial genera, including *Akkermansia*, *Clostridium*, and *Alkaliphilus* (Figure 6C; all *p* < 0.05). Conversely, the abundance of genera *Parabacteroides*, *Lactobacillus*, and *Ruminococcus* was reduced by HF/HF exposure (Figure 6C; all *p* < 0.05). Of note is that maternal (HF/ND: 4.3 ± 0.8%) and post-weaning HF diet (ND/HF: 3 ± 0.5%) both resulted in the reduced abundance in genus *Lactobacillus* compared to the ND/ND group (13.7 ± 2.5%; both *p* < 0.05). The combined HF/HF diet caused the reduction of genus *Lactobacillus* abundance to a greater extent (0.8 ± 0.3%, all *p* < 0.05) (Figure 6D).

**Figure 7:**
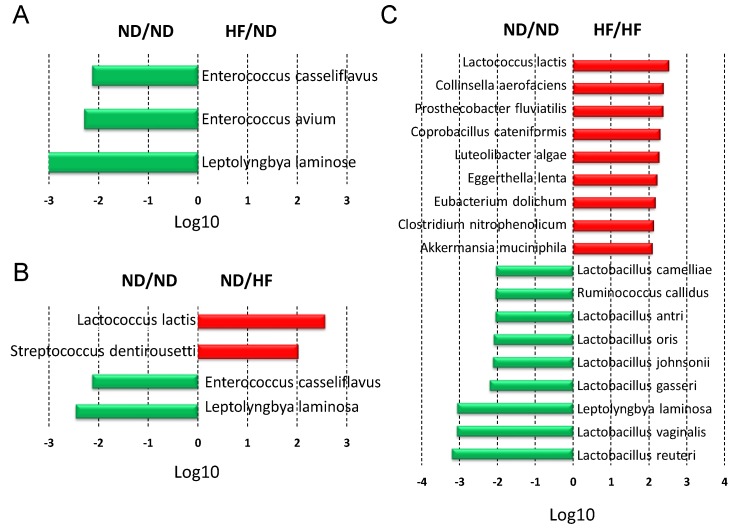
Effect of maternal and post-weaning high-fructose (HF) diet on 16-week-old offspring gut microbiota at the species level. Linear discriminant analysis (LDA), along with effect size measurements, was applied to identify enriched bacterial species. Most enriched and depleted species (LDA score (log10) > 2.0) in the (**A**) HF/ND (red) vs. ND/ND (green), (**B**) ND/HF (red) vs. ND/ND (green), and (**C**) HF/HF (red) vs. ND/ND (green). *n* = 8/group.

The main bacterial species modified by the maternal HF diet were *Leptolyngbya laminosa* (LDA score = −3.1), *Enterococcus avium* (LDA score = −2.3), and *Enterococcus casseliflavus* (LDA score = −2.2) (Figure 7A). The post-weaning HF diet showed an increase in species *Lactococcus lactis* (LDA score = 2.6) and *Streptococcus dentirousetti* (LDA score = 2), and caused a decrease in the species *Leptolyngbya laminosa* (LDA score = −2.5) and *Enterococcus casseliflavus* (LDA score = −2.1) as compared to the ND/ND group (Figure 7B). Of note, there was a remarkable decrease in several species of *Lactobaccilus* in the HF/HF group vs. the ND/ND group (Figure 7C). Conversely, HF/HF diet caused an increase of in species *Akkermansia muciniphila* (LDA score = 2.1).

## 4. Discussion

This study provides a novel insight into the mechanisms responsible for the development of hypertension programmed by maternal and post-weaning HF diet with particular emphasis on gut microbiota-derived metabolites SCFAs and nutrient-sensing signals. The main findings of this study are as follows: (1) combined maternal plus postweaning HF diets induced elevated BP and increased BW and kidney damage in male adult offspring; (2) The combined HF/HF diets caused a rise in BP, which is related to a downregulated AMPK–PGC-1α pathway; (3) The offspring exposed to HF/HF diets had decreased fecal concentrations of propionate and butyrate, decreased renal GPR41 protein levels, and increased renal Oflr78 expression; (4) At 3 weeks of age, the maternal HF diet increased the *Firmicutes* to *Bacteroidetes* ratio and abundance of genus *Clostridium*, and decreased the abundance of genus *Akkermansia* in the gut microbiota in offspring; and (5) The HF/HF diet caused the rise of BP at 16 weeks of age, which was associated with the increased *Firmicutes* to *Bacteroidetes* ratio and reduced abundance in genus *Lactobacillus*. 

In line with previous studies showing that maternal HF intake induces elevated BP in offspring [22,23], our results demonstrated that systolic BP was approximately 5 mmHg higher in the HF/ND group than that in the ND/ND group. Maternal HF diet-induced programmed hypertension may be related to a downregulated AMPK–PGC-1α pathway, an increased *Firmicutes* to *Bacteroidetes* ratio, and a decreased abundance of the genera *Akkermansia* and *Lactobacillus*. Additionally, we found that there is a synergistic effect between maternal and post-weaning HF diet causing a rise in BP and body weight, in support of our previous study showing that effect of maternal nutritional insults on the fetus are not set in stone and can be amplified by changes in the postnatal environment [24,25].

The observed effect of maternal HF diet on BP increase may be related to the inhibition of AMPK–PGC-1α pathway. The interplay between AMPK and other nutrient-sensing signals, driven by maternal nutritional insults, is known to regulate PPARs and their target genes, thus leading to programming of hypertension [17]. AMPKα2 knockout mice expressed activation of the renin-angiotensin system (RAS) to favor the development of hypertension [26]. Also, uni-nephrectomized rats developed hypertension, which was associated with decreased AMPK expression and activation of the RAS [27]. On the contrary, the AMPK activation has been shown to regulate the RAS, resulting in protection from hypertension in different models of programming [28,29]. Recently, AMPK activation has emerged as a reprogramming strategy, via regulating other nutrient-sensing signals like PGC-1α, to protect against hypertension and kidney disease with developmental origins [30]. In the current study, maternal or post-weaning HF diet reduced phosphorylated AMPKα2 and PGC-1α expression. Remarkably, combined maternal and post-weaning HF diets caused the reduction of phosphorylated AMPKα2 and PGC-1α expression to a greater extent in the HF/HF group. These results reconfirmed our previous study showing that combined HF/HF diet-induced hypertension is associated with reduced phosphorylated AMPKα2 and PGC-1α expression. These changes were restored by AMPK activation through resveratrol treatment [7]. These observations suggest that pharmacological therapies aimed at AMPKα2 as a reprogramming intervention to prevent hypertension programmed by maternal HF intake deserve further evaluation.

The results of this study showed that changes of SCFAs and their receptors are another mechanism contributing to HF/HF-induced hypertension. Although maternal HF diet had a neglectable effect on fecal SCFA levels and their receptors, post-weaning HF diet significantly reduced fecal propionate and butyrate concentrations. Propionate and butyrate have been reported to induce vasodilatation via mediating GPR41 and GPR43 receptor [14]. Conversely, acetate is a ligand for Olfr78 to raise BP [14]. Our report showed that combined HF/HF diet decreased fecal propionate and butyrate levels, decreased GPR41 expression, and increased Oflr78 expression in adult offspring kidneys, all of which may favor the development of hypertension. AMPK can be activated by SCFAs, like propionate and butyrate [31,32]. SCFAs have been report to protect against ethanol-induced gut leakiness via AMPK activation [33]. On the other hand, AMPK activation altered microbial populations, which promotes SCFA production [34]. In line with increasing evidence of a link between gut microbiota, SCFAs, and AMPK [35], our study demonstrated that HF/HF-induced hypertension is associated with inactivation of AMPK signaling and the reduction of SCFA production. Additional studies warranted to clarify whether microbiota-derived SCFAs regulate AMPK signaling contributing to hypertension programmed by HF diet. 

Additionally, we observed the major acetate-producing bacteria could be either decreased (e.g., *Lactobacillus*) or increased (e.g., *Clostridiums* and *Akkermansia*) in the HF/HF group with hypertension. Unlike a previous study demonstrating that hypertension-associated dysbiosis is characterized by increases in lactate-producing bacteria [21], results of this study showed that the abundance of genera *Lactobacillus* and *Turicibacter*, which are lactate-producing bacteria, were decreased in the ND/HF and HF/ND-induced hypertension groups. Thus, additional studies are required to clarify whether the imbalance of gut acetate-, butyrate-, and propionate-producing bacterial populations directly contribute to BP control in a variety of programming hypertension models. 

The detrimental effects of HF diet may also relate to alterations in gut microbiota composition. Emerging evidence shows that the development of hypertension is related to gut microbiota dysbiosis in animal models of hypertension [20,21]. Microbiota dysbiosis in early life has deleterious effects and may have long-term consequences leading to many diseases in later life [31]. Our results go beyond previous studies, demonstrating that altered gut microbiota links early-life HF intake to the developmental programming of hypertension. Although the interactions between dietary fat with the gut microbiota have been well explored in human and experimental studies [36], little is known about the impact of maternal fat intake on the offspring gut microbiota. Previous studies showed that maternal HF consumption can alter the offspring microbiome in various animal species [7,37,38]. In line with this, our study demonstrated that maternal HF diet resulted in a considerable impact on the infant microbiota (i.e., 3 weeks of age), as reflected in a higher *Firmicutes* to *Bacteroidetes* ratio, higher abundance of genus *Clostridium*, and lower abundance of genus *Akkermansia*. However, these changes in microbiota compositions seem not persistent until adulthood (i.e., 16 weeks of age). An increased *Firmicutes* to *Bacteroidetes* ratio has been related to obesity in animals fed with saturated fat [36]. Our results go beyond previous reports, showing that mother rats exposed to HF intake caused an increase of the *Firmicutes* to *Bacteroidetes* ratio in their offspring’s microbiota. We found it notable that a certain change in this ratio was persistent until adulthood in the HF/HF group, which had significant BP increases. Given previously published studies using this ratio as a microbial marker for hypertension [20,21], we speculate that this ratio might be a marker to predict hypertension of developmental origins. 

*Akkermansia muciniphila* is the main genus classified in the *Verrucomicrobia* phylum, and recent studies revealed its beneficial effects against obesity and cardiometabolic disease [39]. According to our data, maternal HF diet reduced abundance of genus *Akkermansia* in 3-week-old offspring microbiota. Conflicting with previous reports showing that *Akkermansia muciniphila* abundance inversely correlated with obesity and hypertension [40,41], our results demonstrated that the combined HF/HF diet caused a more than a 100-fold increase of *Akkermansia muciniphila* abundance. Also, HF/HF diet increased abundance of genus *Akkermansia* and phylum *Verrucomicrobia* in offspring microbiota at 16 weeks of age. One possible reason was because we were experimenting in the model of developmental programming, which is more complex than the established disease models. Thus, further studies are needed to elucidate whether *Akkermansia muciniphila* may serve as a microbial marker for hypertension in other developmental programming models. Additionally, we observed that combined HF/HF diets caused a remarkable decrease in abundance of *Lactobacillus*, which is generally considered as a beneficial microbe [42]. Certain probiotic strains like *Lactobacillus* have shown hypotensive effects [43]. As we observed, several *Lactobacillus* spp. were depleted in the HF/HF group, and our previous study demonstrated that maternal *Lactobacillus casei* treatment protected adult offspring against programmed hypertension [15], there is a need to further explore whether early probiotic supplementation may serve as a reprogramming strategy to prevent hypertension programmed by HF/HF diets as well as in other programming models.

Our study has a few limitations. First, we did not examine serial changes in the composition of offspring microbiota. The alterations in gut microbiota we observed in adult offspring may reflect postnatal plasticity rather than programmed processes. Second, we did not analyze other organs controlling BP. The hypertensive effect of HF diet might be attributed to other organs, such as the heart, brain, and vasculature. Third, we employed 16S rRNA gene amplicon analysis to determine proportional changes among bacterial taxonomies. Further studies addressing gene functions contributed by the gut microbiome rather than abundance of taxa to hypertension of developmental origin are required. With the exception of hypertension, maternal HF diet has been used to model other DOHaD-related NCDs [5,44]. It remains to be determined whether changes in microbial composition and their metabolite SCFAs observed in the current study are involved in the pathogenesis of other NCDs. Last, only male offspring were studied in the present study. Given that sex differences appear in gut microbiota and hypertension [19,45], additional studies are required to clarify whether sex-specific interactions between gut microbiota and hypertension exist in mechanisms underlying hypertension programmed by HF diet. 

## 5. Conclusions

In conclusion, several important mechanisms are involved in the development of hypertension programmed by maternal and post-weaning HF diet, including alterations of gut microbiota, SCFAs and their receptors, and nutrient-sensing signals. Targeting AMPK signaling, gut microbiota, and SCFAs might be a reprogramming strategy to reverse the development of hypertension programmed by high fat consumption. Although reprogramming strategies from animal models still await further clinical translation, our findings highlight that pregnant women and children’s caretakers must pay attention to avoid excessive foods that have high fat content.

## Figures and Tables

**Table 1:** Measures of morphological values, blood pressure, and renal function in 16-week-old male offspring exposed to high-fat diet (HF).

Groups	ND/ND	HF/ND	ND/HF	HF/HF
Body weight (BW) (g)	580 ± 8	561 ± 10	680 ± 22 ^a,b^	715 ± 26 ^a,b,c^
Left kidney weight (g)	2.44 ± 0.06	2.17 ± 0.07 ^a^	2.14 ± 0.09 ^a^	2.14 ± 0.09 ^a^
Left kidney weight/100 g BW	0.42 ± 0.01	0.39 ± 0.01	0.32 ± 0.01 ^a,b^	0.30 ± 0.01 ^a,b^
Systolic blood pressure (mm Hg)	142 ± 0	147 ± 1 ^a^	153 ± 1 ^a,b^	168 ± 1 ^a,b,c^
Diastolic blood pressure (mm Hg)	65 ± 2	70 ± 3	73 ± 2 ^a^	76 ± 2 ^a^
Mean arterial pressure (mm Hg)	91 ± 1	96 ± 2 ^a^	99 ± 2 ^a^	107 ± 2 ^a,b,c^
Creatinine (μM)	14.5 ± 0.9	16.2 ± 1.1	17.2 ± 1.1	20 ± 1.8 ^a^

ND/ND, maternal plus post-weaning normal diet; HF/ND, maternal high-fat diet plus post-weaning normal diet; ND/HF, maternal normal diet plus post-weaning high-fat diet; HF/HF, maternal plus post-weaning high-fat diet. BW, body weight; *n* = 8/group; ^a^ *p* < 0.05 vs. ND/ND; ^b^ *p* < 0.05 vs. HF/ND; ^c^ *p* < 0.05 vs. ND/HF.

**Table 2:** Fecal levels of acetate, propionate, and butyrate in in male offspring exposed to high-fat diet (HF) at 16 weeks of age.

Group	ND/ND	HF/ND	ND/HF	HF/HF
Acetate, mM/g feces	3.68 ± 0.13	3.62 ± 0.22	1.34 ± 0.13 ^a,b^	2.46 ± 0.57
Propionate, mM/g feces	0.84 ± 0.06	0.75 ± 0.05	0.25 ± 0.05 ^a,b^	0.47 ± 0.14 ^a^
Butyrate, mM/g feces	1.68 ± 0.21	1.51 ± 0.29	0.22 ± 0.02 ^a,b^	0.27 ± 0.05 ^a,b^

ND/ND, maternal plus post-weaning normal diet; HF/ND, maternal high-fat diet plus post-weaning normal diet; ND/HF, maternal normal diet plus post-weaning high-fat diet; HF/HF, maternal plus post-weaning high-fat diet. BW, body weight; *n* = 8/group; ^a^ *p* < 0.05 vs. ND/ND; ^b^ *p* < 0.05 vs. HF/ND.
